# A Neural Correlate of Predicted and Actual Reward-Value Information in Monkey Pedunculopontine Tegmental and Dorsal Raphe Nucleus during Saccade Tasks

**DOI:** 10.1155/2011/579840

**Published:** 2011-10-12

**Authors:** Ken-ichi Okada, Kae Nakamura, Yasushi Kobayashi

**Affiliations:** ^1^Graduate School of Frontier Biosciences, Osaka University, 1-3 Machikaneyama, Toyonaka 560-8531, Japan; ^2^Department of Physiology, Kansai Medical University, 10-15 Fumizono-cho, Moriguchi City, Osaka 570-8506, Japan; ^3^ATR Computational Neuroscience Laboratories, 2-2-2 Hikaridai, Seika-cho, Kyoto 619-0288, Japan; ^4^PRESTO, Japan Science and Technology Agency (JST), 4-1-8 Honcho Kawaguchi, Saitama 332-0012, Japan

## Abstract

Dopamine, acetylcholine, and serotonin, the main modulators of the central nervous system, have been proposed to play important roles in the execution of movement, control of several forms of attentional behavior, and reinforcement learning. While the response pattern of midbrain dopaminergic neurons and its specific role in reinforcement learning have been revealed, the role of the other neuromodulators remains rather elusive. Here, we review our recent studies using extracellular recording from neurons in the pedunculopontine tegmental nucleus, where many cholinergic neurons exist, and the dorsal raphe nucleus, where many serotonergic neurons exist, while monkeys performed eye movement tasks to obtain different reward values. The firing patterns of these neurons are often tonic throughout the task period, while dopaminergic neurons exhibited a phasic activity pattern to the task event. The different modulation patterns, together with the activity of dopaminergic neurons, reveal dynamic information processing between these different neuromodulator systems.

## 1. Introduction

**Figure 1:**
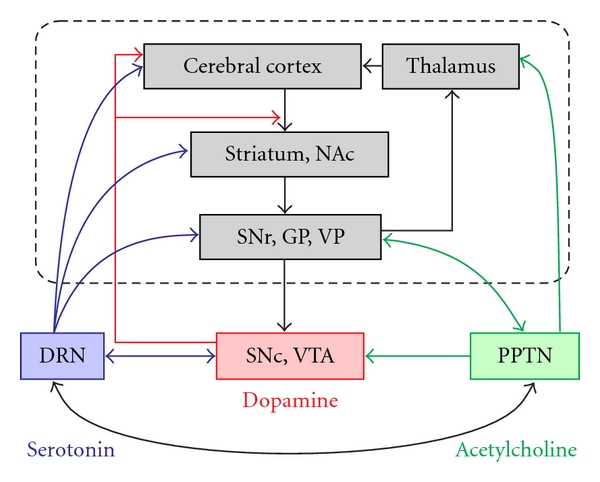
Simplified cortico-basal ganglia circuitry with dopaminergic, cholinergic, and serotonergic innervation. The main cortico-basal ganglia circuit is highlighted by the dashed rectangle and the gray-shaded boxes. Midbrain dopaminergic neurons receive inhibitory input from basal ganglia nuclei and project to the striatum and cerebral cortices. The PPTN and DRN interact with dopaminergic neurons and basal ganglia nuclei. Here, we consider only the major routes by which the basal ganglia and neuromodulators are interconnected. NAc: nucleus accumbens, SNr: substantia nigra pars reticulate, GP: globus pallidus, VP: ventral pallidum, SNc: substantia nigra pars compacta, VTA: ventral tegmental area, DRN: dorsal raphe nucleus, PPTN: pedunculopontine tegmental nucleus.

Reinforcement learning algorithms, originally proposed in the machine learning field, successfully explain various types of adaptive behavioral changes, including the simple classical and operant conditioning of animals [1–6] as well as the complex social and economic behavior of humans [7]. During the reinforcement learning process, subjects choose a behavior that is expected to yield the maximal reward and then revise this prediction on the basis of the reward prediction error, which is the difference between the predicted and actual reward [8]. Numerous neurophysiological studies have shown that midbrain dopaminergic neurons, located in the substantia nigra pars compacta (SNc) and ventral tegmental area (VTA), encode the reward prediction error signal [1, 9–12]. Dopaminergic neurons exhibit phasic burst firing in response to external stimuli and rewards, and the response magnitude alters throughout the course of learning to match the reward prediction error signal [8]. Furthermore, the firing rate of dopaminergic neurons reflects the predicted reward value, which includes the possible reward magnitude, probability of reward delivery, and time delay for receiving the reward [10, 13, 14]. These dopaminergic neurons project to the striatum and cerebral cortices, and the release of dopamine in the projection sites induces synaptic plasticity that corresponds to the revision of reward prediction [6, 15–17] (see Figure 1, red arrows).

Although a large body of experimental evidence has revealed the firing pattern of midbrain dopaminergic neurons and its specific role in reinforcement learning, there is considerable debate about the signal properties of these neurons. First, it was suggested that dopaminergic neurons transmit different types of signals that are related to salient or aversive events [18–22]. Second, in addition to phasic burst firing, a tonic firing pattern has also been observed in dopaminergic neurons [23, 24]. It was suggested that, in the tonic firing mode, dopaminergic neurons maintain a baseline concentration level of dopamine that is vital for motivational behavioral control and to enable the normal functions of the neural circuits. One key issue that remains unclear is the property of the input signal to the dopaminergic neurons. Therefore, several essential elements of reinforcement learning are unsolved, that is, the mechanism for the computation of the reward prediction error and the mechanism for value formation from the interaction of different kinds of information such as the quantity, certainty, and timing of the reward.

Recent pathophysiological and pharmacological studies have suggested that there are mutual interactions between dopamine and other neuromodulators, including acetylcholine, serotonin, and noradrenaline [18, 25–29]. Together with the dopaminergic system, these neuromodulators are proposed to play an important role in gating movement, controlling several forms of attentional behavior [30], and the reinforcement process [28, 31]. The cholinergic pedunculopontine tegmental nucleus (PPTN) and laterodorsal tegmental nucleus (LDT) feed strong excitatory input to midbrain dopaminergic neurons and are reciprocally connected with various basal ganglia nuclei [32] (see Figure 1, green arrows). Additionally, the dorsal raphe nucleus (DRN) is the principal source of serotonergic innervation to the basal ganglia and dopaminergic neurons of rodents [33–37] and primates [38, 39] (see Figure 1, blue arrows). The noradrenergic locus coeruleus (LC) has widely distributed ascending projections to the neocortex [40]. The neurons for these different neuromodulators are plausible candidates as the source of input to dopaminergic neurons and also play an important role in the reinforcement process in parallel with dopaminergic neurons; however, their activity during motivated behavioral tasks remains rather elusive. Thus, in order to understand the network mechanisms underlying reinforcement learning and motivational behavioral control, it is important to elucidate the nature of the signals relayed from the neurons in these principal nuclei of neuromodulators.

We recently recorded the extracellular spike activity of PPTN and DRN neurons in behaving monkeys [41–45]. In this paper, we will compare the activity of neurons in the PPTN/DRN while monkeys performed eye movement tasks to obtain different reward values. We first summarize the growing literature on the PPTN/DRN in relation to the dopaminergic system (Section 2), we then discuss our recent single-unit recording studies from the PPTN/DRN in behaving monkeys (Section 3), and then finally assess the possible mechanisms for reward prediction error computation and its interaction with the motivational signal (Section 4). In short, PPTN and DRN neurons encode the reward prediction and actual reward signals, while dopaminergic neurons encode the reward prediction error signal. The firing patterns of PPTN/DRN neurons are often tonic and sustained throughout the task period, they start shortly after the presentation of the fixation target and are sustained throughout the waiting period and saccade phase until reward delivery, while dopaminergic neurons exhibit a phasic burst to the task event. The reward prediction signals of PPTN/DRN neurons are intermingled with the signals for task motivation.

## 2. Interactions between PPTN, DRN, and Dopaminergic Neurons

### 2.1. Anatomy: Reciprocal Interactions

The PPTN and DRN are heterogeneous nuclei in terms of their neurotransmitters. While the PPTN is the major source of cholinergic projections in the brainstem [46]; it also contains glutamatergic and GABAergic [47–52] as well as dopaminergic [53] and noradrenergic [54] neurons. The DRN is the major source of serotonin in the brain [55], but it also contain neurons with GABA, dopamine, noradrenaline, substance P, and acetylcholine [56].

There are reciprocal anatomical connections between the PPTN, DRN, and dopaminergic systems (Figure 1). Neurons of the PPTN abundantly project to midbrain dopaminergic neurons in the SNc and VTA [57–60]. In rodents, the rostral PPTN projects to the SNc, while the caudal PPTN projects to the VTA [25, 61]. Dopaminergic neurons in the SNc project back to PPTN neurons and excite or inhibit them [62–64], even though the dopaminergic input to PPTN neurons is low compared with the massive cholinergic innervation of dopaminergic neurons. The PPTN also has reciprocal connections with the serotonergic DRN [65–67] and noradrenergic LC [30] monoamine systems. DRN neurons also project to midbrain dopaminergic neurons in the SNc and VTA [33, 36, 68], while dopaminergic neurons also project back to the DRN [69–72].

The PPTN and DRN also have reciprocal interactions with basal ganglia nuclei. The PPTN has massive reciprocal connections with the subthalamic nucleus, globus pallidus, and substantia nigra [73, 74]; thus, it was recently proposed to form a part of the basal ganglia [32]. The DRN projects to the basal ganglia, that is, the striatum, globus pallidus, and substantia nigra [34, 35], as well as to the cerebral cortex and limbic structures [56].

### 2.2. Possible Role of the PPTN/DRN in Controlling the Activity of Dopaminergic Neurons

The PPTN is one of the strongest sources of excitatory input for dopaminergic neurons [75]. PPTN neurons make glutamatergic and cholinergic synaptic connections with dopaminergic neurons [51, 76, 77]. The main effect of acetylcholine on the activity of dopaminergic neurons seems to be excitatory. In rats, electrical stimulation of the PPTN induces a time-locked burst of dopaminergic neurons [24, 78], while chemical or electrical stimulation of the PPTN increases the release of dopamine in the striatum [79–81]. Furthermore, dopaminergic neurons are dysfunctional following excitotoxic lesioning of the PPTN [82]. Other experiments have revealed the receptor level mechanisms underlying the burst firing of dopaminergic neurons induced by acetylcholine from the PPTN and LDT [25, 83, 84]. The burst firing of dopaminergic neurons depends on glutamatergic and cholinergic input [25, 85, 86]. Acetylcholine acts through nicotinic and muscarinic receptors to depolarize dopaminergic neurons and alter their firing pattern [87–90]. Thus, PPTN neuronal activity and acetylcholine provided by PPTN neurons can facilitate the burst firing of dopaminergic neurons [25] and appears to do so via muscarinic [91, 92] and nicotinic [90, 93–95] acetylcholine receptor activation.

Conversely, serotonin can exert either excitatory or inhibitory effects on the activity of midbrain dopaminergic neurons, depending on the subtypes of serotonergic receptors present and the location of the dopaminergic neurons [96]. The main mechanism controlling its action seems to be inhibition by serotonergic 2C/2B receptors [97–100]; however, several serotonergic receptor subtypes facilitate dopamine release [101]. In addition to the direct effect of serotonin via its receptors on dopaminergic neurons, it can also modulate their activity indirectly by modifying GABAergic and glutamatergic input to the VTA and SNc [102, 103].

### 2.3. Possible Role of the PPTN/DRN in Reinforcement Learning

The interactions between the neuromodulator systems are classically associated with wakefulness/sleep control, postural control, and several neuropsychiatric disorders [27, 66, 104, 105].

In addition to these numerous functional roles, recent studies have suggested that the PPTN is critically involved in various reinforcement processes [106–110]. Lesioning of the PPTN before operant training disrupted the acquisition of the self-administration response, while lesioning after training did not [111, 112]. Lesioning, stimulation, and reversible inactivation of the PPTN impaired the performance in several conditioned task behaviors, but they did not change simple behavior, including locomotion, feeding, and lever pressing [113–115].

Similarly, several lines of evidence suggest that the entire raphe or serotonin regulates motivated behavior [28, 31, 116–123]. The depletion of serotonin induces impulsive behavior, which might reflect a deficit of the valuation system. The systemic or local depletion of serotonin renders an animal likely to choose a small but immediate reward rather than a large but delayed reward [124–131]. The human DRN was activated when subjects learned to obtain large future rewards [119]. Long-lasting DRN activity may also have other functions because impulsivity has been associated with other serotonin-related behavioral tendencies such as aggression [132, 133] and obsession [134].

## 3. Responses of PPTN/DRN Neurons in Two-Valued Reward Saccade Tasks

Thus, abundant anatomical, electrophysiological, and pharmacological studies of slice and whole animal preparations indicate that PPTN/DRN neurons provide mutual inputs to dopaminergic neurons and basal ganglia nuclei and play an important role in reinforcement learning. However, the precise mechanism by which PPTN/DRN neurons cause these effects is unknown, partly because only a few studies have examined the activity of PPTN/DRN neurons during motivated behavioral tasks.

Classically, electrophysiological studies of PPTN neurons have shown their relationship with the sleep-wake cycle and locomotion [30]. Further, in a pioneering study of operant conditioned cats, PPTN neurons relayed either a reward or salient event signal by phasic firing [135]. A recent study in rats showed that the reward-related activity of PPTN neurons was affected by changes in the reward context [136]. Other studies have reported that PPTN neurons encoded the sensory or motor rather than reward information of task events in rats [137] and monkeys [138].

For DRN neurons, electrophysiological studies have mainly focused on the sleep-wake cycle and motor behavior [139], and recent studies in rats reported that DRN neurons showed transient changes in activity to sensorimotor events, including reward [140] and aversive foot shocks [141]. Recent studies in rats also reported that the efflux of serotonin was enhanced [142], and the tonic firing of DRN neurons was increased [143] while rats waited for a reward, which was related to their waiting behavior.

To examine the role of the PPTN/DRN in reward prediction error computation and adaptive behavioural control, we recorded the extracellular spike activity of PPTN, DRN, and putative dopaminergic neurons in monkeys performing saccade tasks to obtain a juice reward [41–45]. We used two-valued reward saccade tasks, that is, visually-guided and memory-guided saccade tasks, which are comparable to those used for electrophysiological recordings from basal ganglia nuclei and dopaminergic neurons. In the visually-guided saccade task, the animal maintained fixation on a central fixation target, and, immediately after the peripheral target appeared, it made a horizontal saccade. In the memory-guided saccade task, the animal made a saccade to a flashed target location after some delay.

**Figure 2:**
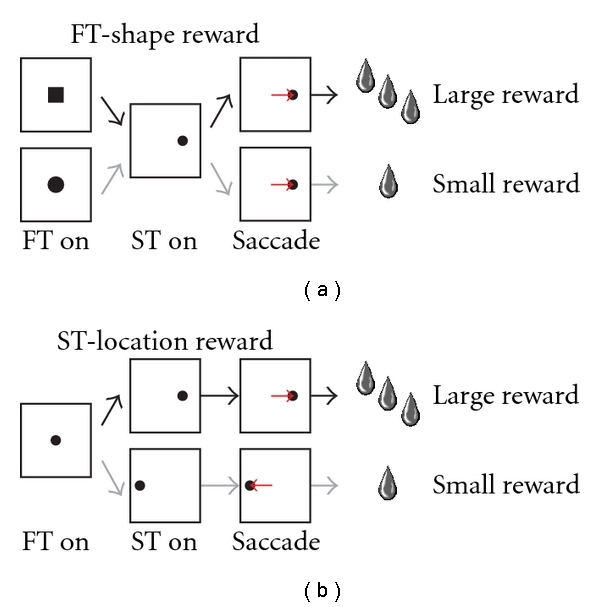
Schematic diagrams for the two-valued reward saccade tasks. (a) The reward magnitude was cued by the shape of the initial central fixation target (square or circle) for recordings from PPTN neurons. (b) The location of the saccade target (left or right) was associated with large or small rewards, respectively, in recordings from DRN neurons. FT: fixation target, ST: saccade target.

To examine (1) the effect of the predicted reward value and (2) the effect of error in reward prediction on neuronal activity, we made two modifications to the tasks. First, in order to examine the effect of reward prediction, we made these saccade tasks two valued so that the reward magnitude (large or small) was cued by the property of the visual target (shape or location) in each trial. For recordings from PPTN neurons [42], the reward magnitude was cued by the shape of the initial central fixation target (Figure 2(a), square or circle). For recordings from DRN neurons and putative dopaminergic neurons in the SNc [44, 45], the location of the saccade target (left or right) was associated with large or small rewards, respectively (Figure 2(b)). In these conditions, the monkeys learned the relationship between the property of the cue and the reward magnitude, and the behavior of the monkeys was influenced by their expectation of the reward value.

Second, in order to examine the effect of the reward prediction error which was the difference between the actual given reward and the predicted reward, we changed the contingency between the cue property and the reward value. Specifically, the cue property (either fixation target shape or saccade target location) and the reward value contingency was constant for more than 20 consecutive trials, called a block. Because of the block design, once a block was started, the animal knew which cue property generated the largest reward, even before cue presentation. Then the contingency between the cue property and the reward value was switched without any additional cue; therefore, the animal only received an unexpected reward magnitude on the very first trial after contingency reversal.

For extracellular recording, the locations of the PPTN and DRN were estimated using magnetic resonance imaging and later verified histologically. Details of recording sites of the PPTN and DRN are shown in Figure  1 of Okada et al. [42] and Figure  1 of Nakamura et al. [44], respectively. Correct placement of the recording electrode was also confirmed by monitoring the neuronal activity in the surrounding structures, including the superior and inferior colliculi. For recordings from PPTN neurons, high-frequency tonic fiber activity in the cerebellar peduncle, close to the PPTN, was used as a landmark. For recordings from the DRN, which has a more medial location than the PPTN, the trochlear nucleus is the most prominent landmark in monkeys [144].

To record from putative dopaminergic neurons, we searched in and around the SNc. Dopaminergic neurons were identified by their irregular and tonic firing at ~5 spikes/s with broad spike potentials. The recording sites were estimated using magnetic resonance imaging and later verified histologically. In this experiment, we focused on those dopaminergic neurons that responded to reward-predicting stimuli with phasic excitation.

As noted above, although the PPTN and the DRN are centers of cholinergic and serotonergic neurons; respectively, they also contain neurons with other neurotransmitters. This heterogeneity poses a challenge to relate electrophysiological studies of PPTN/DRN neurons to their neurochemical identity. It was suggested that there are 2 types of neurons in slice preparations of the rat PPTN that generated broad and brief action potentials [145]. Recent extracellular recording studies also reported *neurons* that generated broad and brief action potentials; however, they exhibited a unimodal distribution and could not be classified into groups [41, 138]. For the DRN, previous studies estimated that a substantial proportion of DRN neurons are serotonergic: ~30% in rats [146], 70% of medium-sized DRN neurons in cats [55, 147], and 70% in humans [148]. Note that, in addition to serotonin, the DRN includes neurons with many kinds of neurotransmitters such as GABA, glutamate, and dopamine [56]. However, there are no reliable electrophysiological criteria (such as the baseline firing rate, spike shape, and spiking regularity) to identify the neurotransmitter of the recorded neuron. Therefore, we studied all well-isolated neurons in the PPTN/DRN whose activity changed during saccade tasks, rather than choosing neurons with specific electrophysiological properties.

### 3.1. Neuronal Activity of the PPTN

We recorded the extracellular spike activity of PPTN neurons during the performance of the two-valued saccade tasks in monkeys [42]. These tasks were comparable to those used in recordings from basal ganglia nuclei and dopaminergic neurons in which the shape of the fixation target (square or circle) indicated the reward magnitude (large or small, Figure 2(a)). We recorded a population of PPTN neurons that exhibited significant responses to one or more task events, including reward delivery, visual stimulus presentation, and saccade execution (153/185, 83%). The responses showed a rich variety of patterns: some exhibited a phasic response to task events, others exhibited tonic changes in activity throughout the trial, and we also observed a combination of these phasic and tonic responses.

In this section, we will describe the activity modulation of PPTN neurons for (1) the prediction of reward magnitude, (2) motivation to perform the task, and (3) actual reward magnitude. In short, two groups of PPTN neurons showed reward magnitude-dependent response modulation. A subset of neurons exhibited increased activity around the time of the onset of the fixation target that was sustained until the end of the trial, with a significant dependency on the magnitude of the predicted reward (*fixation target* neurons, Section 3.1.1), while the other neurons exhibited a phasic increase in activity only around the time of reward delivery, with a significant dependency on the reward magnitude of the current reward (*reward delivery* neurons, Section 3.1.3). All of these observed features of PPTN neuronal activity are suitable for its possible role in reward prediction error computation and appropriate action selection in a given situation.

#### 3.1.1. Effect of the Predicted Reward Value on the Activity of PPTN Neurons

**Figure 3:**
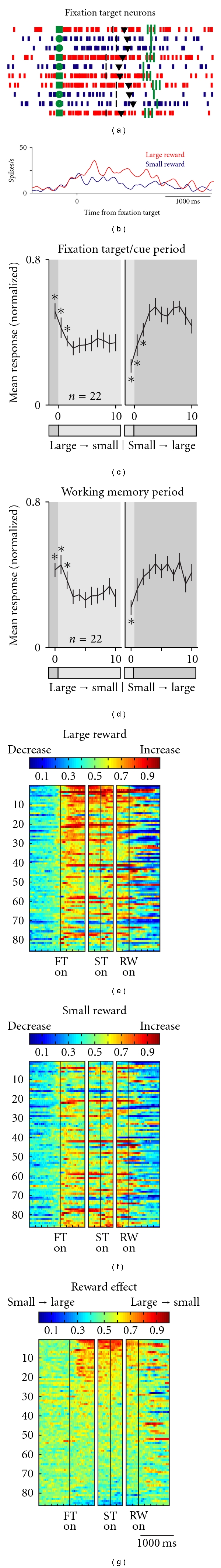
Activity of fixation target neurons of the PPTN for the saccade task. (a, b) A rastergram and peritask event spike density function for the activity of a representative fixation target neuron over 10 successive trials, aligned to the onset of the fixation target. The red and blue rasters (a) and traces (b) indicate large and small reward trials, respectively. In (a), the green squares and circles indicate fixation target onset, the black bars indicate the onset of the saccade target, the black triangles indicate saccade onset, and the green lines indicate the times at which the large (3 bars) and small (1 bar) rewards were delivered. (c) Responses of fixation target neurons to fixation target (squares and circles) presentation (mean response of 200–600 ms after fixation target onset, fixation target/cue period) after reversal of cue-reward contingency. The left panel shows the large-to-small reward reversal, and the right panel shows the small-to-large reward reversal. Large-reward trials are indicated by the dark gray bars, while small-reward trials are indicated by the clear areas. Shown are the mean and standard error of the mean (SEM) of the normalized neuronal activity for the *n*th trial after contingency reversal. The asterisks (*) indicate the activity that was significantly different from the activity during the last 5 trials of the block with the reversed contingency (*P* < 0.01, Mann-Whitney *U* test). (d) Similar to (c) but for the responses after fixation target offset (working memory period, 200–600 ms after fixation target offset). (e–g) The activity of each fixation responsive neuron is presented as a row of pixels (*n* = 86). (e, f) Changes in the neuronal firing rate from baseline are compared in the large- (e) and small- (f) reward trials. The color of each pixel indicates the ROC value based on the comparison of the firing rate between a control period just before fixation onset (400-ms duration) and a test window centered on the pixel (100-ms duration). Warm colors (ROC > 0.5) indicate increases in the firing rate relative to the control period, whereas cool colors (ROC < 0.5) indicate decreases in the firing rate. (g) Changes in reward-dependent modulation. The ROC value of each pixel was based on the comparison of the firing rate between the large- and small-reward trials. Warm colors (ROC > 0.5) indicate higher firing rates in the large-reward trials than in the small ones. In these 3 panels (e–g), the neurons have been sorted in order of their ROC values for the reward prediction effect during the task period. FTon: fixation target onset; STon: saccade target onset; RWon: reward onset. (Modified from [42].)

A subset of PPTN neurons exhibited increased activity around the time of the onset of the fixation target that was sustained until the end of the trial, with a significant dependency on the magnitude of the predicted reward (*fixation target* neurons, *N* = 30, Figure 3). Figures 3(a) and 3(b) show raster displays and spike density functions for a representative fixation target neuron. This neuron showed elevated firing throughout the trial that was greater when the cued reward was large; compare the red raster lines and traces (large reward) with the blue ones (small rewards). Differences in the responses to the large and small reward cues generally began to emerge at ~100 ms after the cue was presented. These differential responses extended throughout the working memory period following the offset of the fixation target/cue and lasted until, and even after, reward delivery (green bars), and they were almost unaffected by other task events, such as the onset of the peripheral saccade target (black bars) and the saccade to the saccade target (black triangles). Note that there were nondifferential responses before the onset of the fixation target, presumably in anticipation of its appearance. In the next section, we will discuss the relationship between these nondifferential responses and the monkeys' motivation to perform the task. We used multiple analytical approaches, including receiver operating characteristic (ROC) analysis, mutual information, and correlation analyses, and all analyses consistently proved the dependency of the neuronal activity on the magnitude of the predicted reward [42]. Because some fixation target neurons maintained these differences in response even after reward delivery, we also tested their response to free-reward delivery, in which the large reward was given unexpectedly during the intertrial intervals. All of the tested fixation target neurons were totally unresponsive to free-reward delivery, consistent with the view that these neurons encode the predicted reward value instead of the actual reward or reward prediction error signals.

The tonic modulations in activity during the task period, as shown in the example neuron in Figures 3(a) and 3(b), were commonly observed in the PPTN neurons (*N* = 86, Figures 3(e)–3(g)). After fixation target onset, but before reward delivery, approximately one-third of fixation target-responsive PPTN neurons showed significant reward-dependent modulation, with most of the neurons firing more strongly for large- than small-reward trials (*N* = 30, Figure 3(g)). There was a small population of neurons that showed a weak negative reward magnitude dependency in which the response was smaller during the large-reward trials (*N* = 6). For each neuron, the changes in activity during the task period tended to increase and be sustained during large- and small-reward trials but was greater during large-reward trials, thus leading to the differences in activity between the two reward conditions (Figures 3(e) and 3(f)).

Further insights were obtained by recording the activity in a contingency reversal paradigm, in which the meaning of the fixation target/cue was suddenly reversed during neuronal recording (Figures 3(c) and 3(d)). As a result of contingency reversal, there was a discrepancy between the predicted and actual reward, at least during the first trial, and we examined the trial-by-trial responses of the fixation target neurons around the contingency reversal period. The responses of the fixation target neurons during the fixation target period and the subsequent working memory period clearly reflected the contingency reversal with a delay of one trial. In the first reversed contingency trial, the animals could not predict the correct reward magnitude because they were unaware of the contingency reversal, and the target/cue and working memory period responses did not immediately follow the contingency reversal. The net result was that, by the second trial after contingency reversal, the cue predicting the larger reward was again associated with the higher discharge rate (i.e., one-trial learning).

#### 3.1.2. Correlation of Fixation Target Response with Behavioral Performance

As shown above, a population of PPTN neurons showed tonic activity changes throughout the task period, and a subset showed reward value-dependent activity modulation. We then examined the relationship between the task- and reward-related modulations.

**Figure 4:**
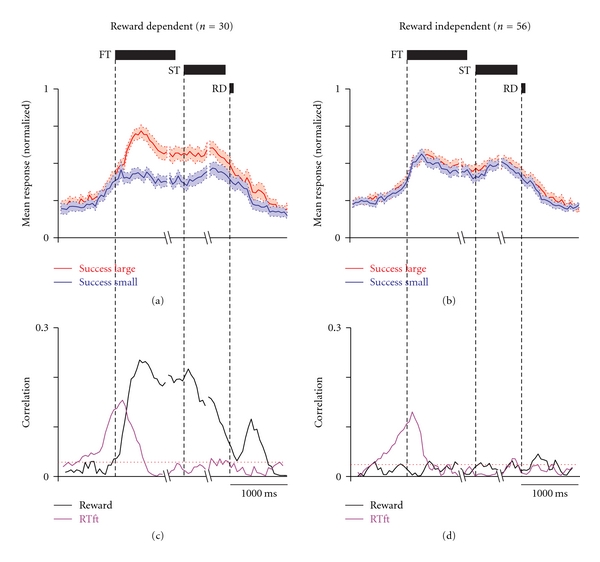
Correlations between PPTN neuronal responses with reward value and task performance. (a, b) Population spike density function of reward magnitude-dependent (a) and -independent (b) fixation target-responsive neurons averaged for large- (red) and small- (blue) reward trials, aligned to fixation target onset, saccade target onset, and reward delivery. The spike density is the population average normalized for the peaks of the individual neurons. The thick lines indicate the mean normalized activity, and the light-shaded areas are ± 1 SEM. (c, d) Correlation coefficient (absolute value) plots of the neuronal responses shown in (a) and (b) with the reaction time to fixate upon the fixation target (purple) and the reward magnitude (black). The horizontal dotted red line indicates the significance level (*P* = 0.05) of the correlations. FT: fixation target, ST: saccade target, RD: reward delivery. (Modified from [42].)

The population-averaged normalized activity of PPTN neurons is shown in Figure 4, separately from the reward-related modulation patterns. As shown by the normalized activity modulation of each neuron in Figure 3, reward value-dependent and -independent neurons showed elevated activity during the task period. The correlation between the neuronal activity and reward value was significant for reward value-dependent neurons, peaked after the presentation of the fixation target and was sustained during the task period (Figure 4(c), black trace). Conversely, there was almost no correlation for reward value-independent neurons (Figure 4(d), black trace).

The increase in activity started even before the onset of the fixation target, presumably in anticipation of its appearance. Interestingly, the responses of the reward magnitude-independent neurons during the precue period were identical to those of the reward magnitude-dependent fixation target neurons (Figures 4(a) and 4(b)). To test whether the PPTN neurons encoded the motivation to fixate on the target, we analyzed the relationship between the activity during the precue period and the reaction time to fixate upon the initial fixation target (RTft).

Now, if the neurons encoded motivation in an integrated manner, then the neurons that showed reward value-dependent modulation should also show behavioral performance dependency, whereas neurons that showed no reward value dependency should also show no behavioral performance dependency. Conversely, if the neurons encoded the motivation to fixate on the target and the motivation to get the reward in an independent manner, then there should be no systematic relationship between behavioral performance dependency and reward value dependency.

The neuronal activity was correlated with RTft in a time-dependent manner in the reward magnitude-dependent and -independent neuronal groups. This correlation became significant during the precue period, peaked shortly after the presentation of the fixation target, and declined back to baseline during the cue period (Figures 4(c) and 4(d), purple trace). Altogether, the reward magnitude-independent neurons shared the component for the response correlation related to the anticipation of cue onset with the reward magnitude-dependent neurons. This finding indicates that the reward magnitude-independent neurons signal the early component of the motivational drive to fixate on the fixation target in an almost equal manner to that of the reward magnitude-dependent fixation neurons.

#### 3.1.3. Effect of the Received Reward Value on the Activity of PPTN Neurons

**Figure 5:**
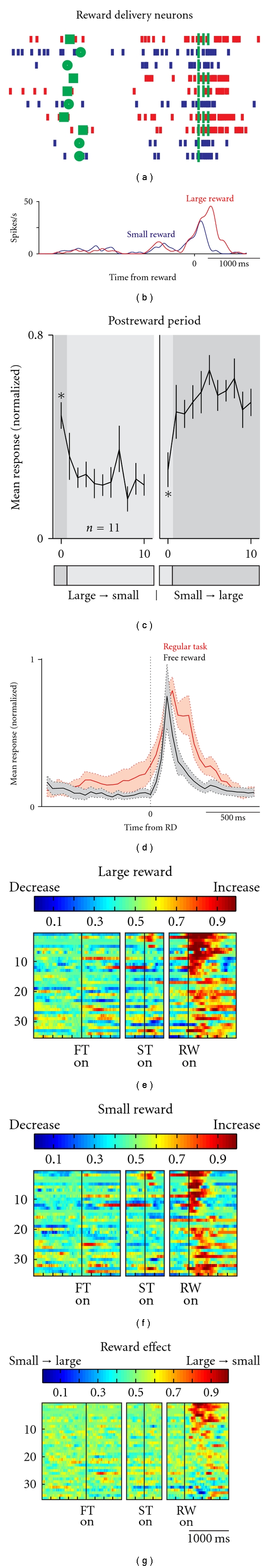
Activity of reward delivery neurons of the PPTN for the saccade task. (a, b) A rastergram and peritask event spike density function for the activity of a representative reward delivery neuron over 10 successive trials, aligned to reward delivery. (c) Responses of the reward delivery neurons to reward delivery of large and small rewards after the reversal of cue-reward contingency. (d) Population response of reward delivery neurons to free (black) and large (red) rewards. The responses represent the average firing rate normalized for the peak responses of the individual neurons (*n* = 9). The thick lines indicate the mean normalized activity, and the light-shaded areas are ± 1 SEM. (e–g) The activity of each reward-responsive neuron is presented as a row of pixels (*n* = 35). (e, f) Changes in the neuronal firing rate from baseline are compared in the large- (e) and small- (f) reward trials. (g) Changes in reward-dependent modulation. In these 3 panels (e–g), the neurons have been sorted in order of their ROC values for the reward effect during the postreward delivery period. FTon: fixation target onset; STon: saccade target onset; RWon: reward onset. (Modified from [42].)

Another group of PPTN neurons exhibited a phasic response to reward delivery, with a significant dependency on the magnitude of the delivered reward (*reward delivery* neurons, *N* = 15). In contrast to the tonic activity of the fixation target neurons, the reward delivery neurons exhibited a transient response, reaching a peak discharge rate shortly after reward delivery and then rapidly declining back to baseline (Figures 5(a) and 5(b)); these were almost unresponsive during the target/cue and working memory periods. In the trial with a larger reward, the discharge rate of the transient response reached a higher peak at a slightly later time and took a few hundred milliseconds longer to decay back to baseline than during the small-reward trials. Similar to the fixation target neurons, approximately half of the reward delivery neurons showed small nondifferential responses, even before reward delivery, presumably in anticipation of the timing of the reward.

After actual reward delivery, approximately half of the reward-responsive PPTN neurons showed significant positive-reward-dependent modulation and fired more strongly during large- than small-reward trials (15/35, Figures 5(e)–5(g)). There was a small population of neurons that showed a weak negative-reward-magnitude dependency (*N* = 5). For each neuron, the changes in activity after reward delivery tended to increase during the large- and small-reward trials.

During the contingency reversal paradigm, there was a discrepancy between the predicted and actual reward. The responses of the reward delivery neurons changed immediately after the contingency reversal, so that larger rewards were still associated with larger neuronal responses, even on the first trial in which the monkeys predicted the small rewards (Figure 5(c)). Therefore, the reward delivery neurons convey information about the magnitude of the actual given reward, regardless of the monkeys' prediction. We also tested the responses to free-reward delivery, and all of the tested reward delivery neurons responded briskly to the task- and free-reward delivery. The fact that the reward delivery neurons responded to the task and free rewards, given in either an expected or unexpected manner, suggests that reward delivery neurons encode the actual reward magnitude. This is fundamentally different from the reward response of dopaminergic neurons that exhibited burst firing only to an unexpectedly given reward and showed no response to the fully predicted reward (reward prediction error, see also Figure 8) [9, 149].

Overall, two different groups of PPTN neurons encode the reward prediction and actual reward signals, both of which are necessary for the computation of the reward prediction error signal in dopaminergic neurons. The reward prediction signal is encoded by the sustained tonic firing of one group of PPTN neurons (Figure 3) and is sometimes intermingled with the task motivation signal (Figure 4). The actual reward signal is encoded by the phasic response of the other group of PPTN neurons (Figure 5).

### 3.2. Neuronal Activity of the DRN

We also recorded extracellular spike activity from the neurons in the monkey DRN during the two-valued saccade tasks [44, 45]. The tasks were comparable to those used for the PPTN recordings, except that the location of the saccade target (left or right) indicated the reward magnitude (large or small, Figure 2(b)). We observed that, like PPTN neurons, DRN neurons also exhibited tonic changes in activity that would be ideal to encode sustained aspects of motivated behavior such as the predictive state of the upcoming reward. Detailed analyses indicated that a group of DRN neurons did indeed keep track of the predicted and/or given reward value.

#### 3.2.1. Effect of the Predicted and Received Reward Value on the Activity of DRN Neurons

**Figure 6:**
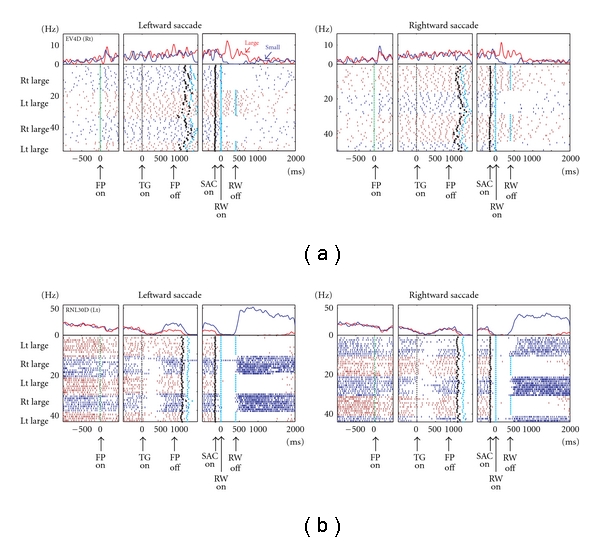
Activity of two example DRN neurons for the saccade task. For each neuron, (a) and (b), the rasters and histograms for the leftward and rightward saccades are shown separately. The changes in their firing rates are shown by the peritask event spike density function at the top. The activity in the large- and small-reward trials is shown in red and blue, respectively. The data are shown in 3 sections: the left section is aligned to the time of fixation point onset (FPon), the middle section is aligned to target onset (TGon) and fixation point offset (FPoff), and the right section is aligned to reward onset (RWon). Note that the reward offset (RWoff) applies only to the large-reward trials. The black dots indicate saccade onset (SACon), and the light blue dots indicate reward onset and offset. (Modified from [44].)

DRN neurons exhibited task-related activity that was modulated by the reward value.  Figure 6(a) shows a representative example. The neuron exhibited an increase in activity after the onset of the fixation point (FPon) followed by regular and tonic firing until reward onset. The activity further increased after the onset of a large reward but ceased after the onset of a small reward and lasted for more than 800 ms after reward onset. A subset of neurons, an example of which is shown in Figure 6(b), exhibited the opposite pattern; that is, the neuron showed small reward-dominant post-reward activity that lasted until the start of the next trial. In some neurons, reward value-dependent modulation was also observed during the delay period, *before *reward onset, presumably reflecting the monkeys' prediction of the reward. The neuron in Figure 6(b) exhibited stronger delay activity during small-reward trials than during large-reward trials, but only when leftward saccades were required. However, note that such directional selectivity was relatively rare among DRN neurons, and many neurons showed reward value-dependent modulation regardless of the direction of the saccade. 

**Figure 7:**
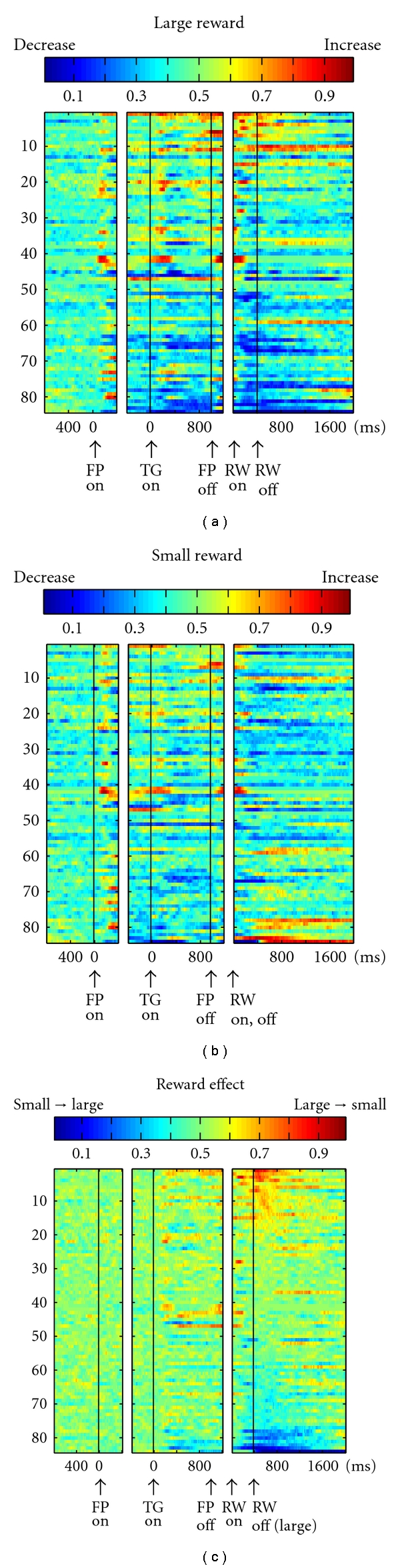
Population activity of DRN neurons. The activity of each neuron is presented as a row of pixels (*n* = 84). (a, b) Changes in the neuronal firing rate from baseline are compared in the large- (a) and small- (b) reward trials. The color of each pixel indicates the ROC value based on the comparison of the firing rate between a control period just before fixation onset (400-ms duration) and a test window centered on the pixel (100-ms duration). Warm colors (ROC > 0.5) indicate increases in the firing rate relative to the control period, whereas cool colors (ROC < 0.5) indicate decreases in the firing rate. (c) Changes in reward-dependent modulation. The ROC value of each pixel was based on the comparison of the firing rate between the large- and small-reward trials. Warm colors (ROC > 0.5) indicate higher firing rates during the large-reward trials than during the small ones. In all panels (a–c), the neurons have been sorted in order of their ROC values for the reward effect during the postreward (400–800 ms) period (c). FPon: fixation point onset, TGon: target onset, FPoff: fixation point offset, RWon and off: reward onset and offset. (Modified from [44].)

The reward-dependent modulations in activity before and after reward delivery, as shown in the example neurons in Figure 6, were commonly observed in DRN neurons (Figure 7). After target onset, but before reward delivery, approximately one-quarter of all analyzed DRN neurons showed significant reward-dependent modulation, with most of the neurons firing more strongly for large than small reward trials (Figure 7(c)). After reward delivery, more than 40% of neurons exhibited reward-dependent modulation, with half of them preferring large rewards and the other half preferring small rewards.

Note that there was a notable difference in the reward-dependent modulation between the pre- and postreward periods. For each neuron, the changes in activity during the prereward period, compared with the baseline activity, tended to be in the same direction during large- and small-reward trials but tended to be greater during large-reward trials, thus, leading to differences in the activity between the two reward conditions (Figures 7(a) and 7(b)). On the contrary, the changes in activity during the postreward period, compared with the baseline activity, tended to be in the opposite direction. For example, for the neuron shown in Figure 6(a), the prereward activity increased compared with the baseline during large- and small-reward trials. However, the postreward activity increased during large-reward trials, but it was inhibited during small-reward trials. Such a distinct effect on modulation indicates a different source for the modulation of DRN neuronal activity before and after reward delivery.

**Figure 8:**
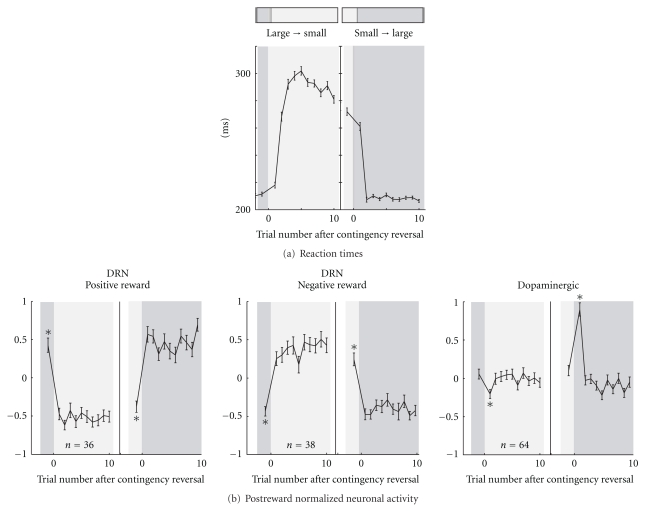
Changes in the reaction times and activity of DRN and putative dopaminergic neurons with reward contingency reversal. The reaction times (a) and normalized neuronal activity during the postreward period of DRN (400–800 ms after reward onset) and putative dopaminergic neurons (0–400 ms after reward onset) are plotted. In (b), the data are shown for DRN neurons with a large-reward preference (left), DRN neurons with a small-reward preference (middle), and putative dopaminergic neurons (right). Error bars, SEM. (Modified from [44].)

While recording from DRN neurons, the contingency between the target position and reward value was fixed during one block of trials but was then reversed with no external cue. This allowed us to examine how the monkeys' performance and neuronal activity changed to the new position-reward contingency. The saccadic reaction times changed quickly after the reversal of the position-reward contingency (Figure 8(a)). We, therefore, examined the time course of the changes in the mean normalized firing rates for DRN neurons (400–800 ms after reward onset) and for the putative dopaminergic neurons (0–400 ms after reward onset) as a function of the trial number after reversal.

There was a striking difference between the DRN neurons and dopaminergic neurons in their postreward activity. The activity of DRN neurons faithfully followed the size of the reward (Figure 8(b), left and middle). In other words, DRN neurons reliably coded the value of the received reward whether or not it was expected. In contrast, the activity of the dopaminergic neurons only changed transiently during the first trial and, thereafter, returned to a level close to baseline activity (Figure 8(b), right). Specifically, dopaminergic neurons decreased their postreward activity for large-to-small reward reversals and increased their activity for small-to-large reversals. These transient changes in postreward activity represent the “reward prediction error,” which is the difference between the value of the predicted (e.g., small reward) and the actual rewards (e.g., large reward). This progression in the postreward activity of dopaminergic neurons is consistent with the findings of other studies [9, 149]. Thus, the results indicate that DRN neurons encode the actual reward value and not the reward prediction error.

#### 3.2.2. Coding of the Task Reward Value in the DRN

As shown in Figure 6, the response of the DRN neurons often took the form of tonic activity changes throughout multiple task phases. Such type of activity would be ideal to encode sustained aspects of motivated behavior such as the state of expectation for the upcoming reward.

To test this hypothesis, we analyzed the relationship between the tonic activity during the fixation period and the differential responses to reward cues and actual rewards. Note that during the fixation period (before target onset), the exact reward value the animal would receive for that trial was as yet unknown (Figure 2(b)). However, the overall value of the behavioral task would be between the large- and small-reward value, which may be expressed by the neuronal firing rate during the fixation period. Now, if the neurons encoded behavioral tasks primarily in terms of their reward value throughout a trial, then the neurons that were excited during the fixation period should preferentially be excited by the reward cues and the actual reward, whereas the neurons that were inhibited during the fixation period should be preferentially inhibited by the reward cues and the actual reward. On the contrary, if the neurons encoded the information (including the reward value) during the fixation period and after the reward cue and reward delivery in an independent manner, then there should be no systematic relationship between the fixation and reward-related activity.

**Figure 9:**
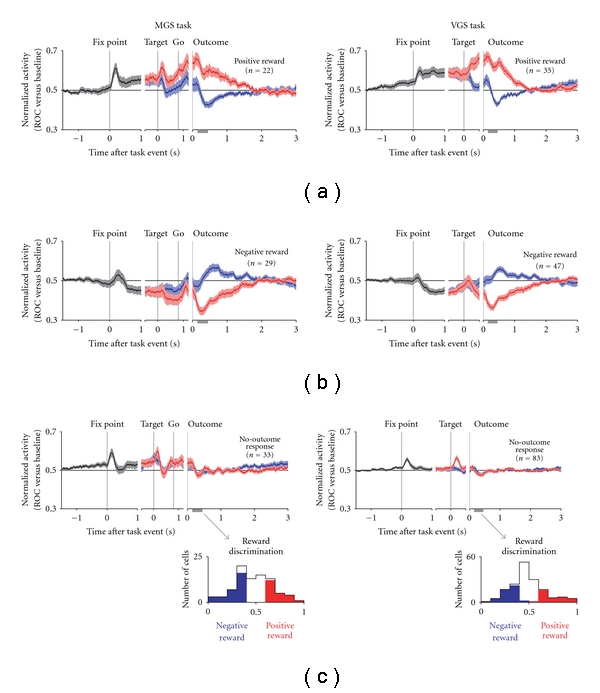
Population-averaged activity of DRN neurons separated by their reward signals in response to the outcome. (a–c) Normalized activity is shown for the memory-guided saccade task (MGS, left) and visually-guided saccade task (VGS, right), shown separately for positive-reward neurons (a, top), negative-reward neurons (b, middle), and no-outcome response neurons (c, bottom). The colors indicate the average of all trials (black), large-reward trials (red), and small-reward trials (blue). The neurons were sorted into these categories on the basis of significant reward discrimination after outcome onset (gray bar on the *x*-axis; *P* < 0.05, Wilcoxon rank-sum test). The histograms below (c) show the reward discrimination for each neuron, with the colors indicating positive-reward neurons (red) and negative-reward neurons (blue). For the plots of normalized activity, the activity of each neuron was normalized by computing its ROC area versus baseline activity during the intertrial interval. The thick lines indicate the mean normalized activity, and the light shaded areas are ±1 SEM. (Modified from [45].)

The population-averaged normalized activity of DRN neurons is shown in Figure 9 and separately for neurons with positive (Figure 9(a)), negative (Figure 9(b)), or no significant reward signals (Figure 9(c)) in response to reward delivery. Neurons with positive-reward signals for reward delivery (stronger activity for a large reward than for a small reward) had elevated activity during the fixation period (Figure 9(a)). If the large-reward target appeared, their activity was elevated further, whereas if the small-reward target appeared, they returned to near the baseline. Neurons with negative-reward signals (stronger activity for a small reward than for a large reward) had suppressed activity during the fixation period (Figure 9(b)). If the large-reward target appeared, their activity was further suppressed, whereas, if the small-reward target appeared, they returned to near the baseline. Neurons with no significant reward signals had a tendency for phasic responses to the fixation and saccade targets and slightly elevated activity during the fixation period (Figure 9(c)). Further analyses revealed that neurons with stronger task coding, that is, changes in their fixation period activity, also had stronger reward coding, that is, different activity between the large- and small-reward trials. Collectively, such equivalent changes in activity between the fixation and postreward periods suggest that the level of DRN activity continually tracks the predicted value. 

## 4. Circuit Mechanisms for the Computation of the Reward Prediction Error Signal

### 4.1. Summary of the Response Patterns of PPTN/DRN Neurons

**Figure 10:**
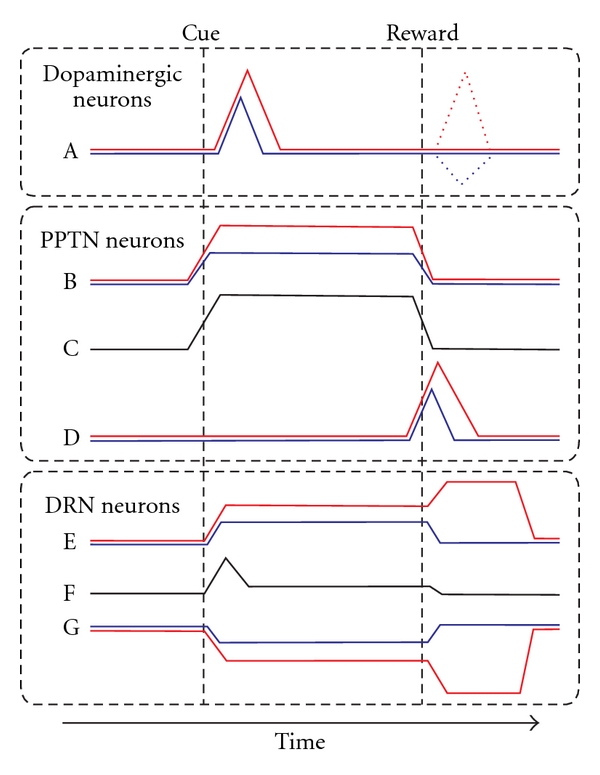
Schematic drawing of the activity changes of dopaminergic, PPTN, and DRN neurons for the two-valued saccade task. Cue and reward indicate the timing of reward-cue presentation (either fixation target shape or saccade target location) and large- and small-reward delivery, respectively. The colors indicate the responses in the large-reward trials (red) and small-reward trials (blue) and the responses of neurons with no significant reward modulation (black). (A) Dopaminergic neurons exhibited phasic burst firing to a reward-predictive cue and an unexpected reward (dashed lines). (B, D) Two different groups of PPTN neurons exhibited a tonic reward prediction response (B) and a phasic actual reward response (D). (C) PPTN neurons with no significant reward modulation often exhibited tonic activity during the task period. (E, G) DRN neurons exhibited correlated central fixation and reward modulation, preferring either larger (E) or smaller rewards (G). (F) DRN neurons with no significant reward modulation often exhibited a phasic response to target presentation.

Here we summarize and compare the temporal activity patterns of the dopaminergic, PPTN, and DRN neurons to the presentation of the reward-predicting cue and reward delivery in the two-valued reward task (Figure 10).

In the earlier phases of the trial, the reward-predicting cue was presented. The dopaminergic neurons then exhibited a phasic burst of activity. The magnitude of their response was correlated with the predicted reward value, such that greater firing occurred in response to more valuable cues (Figure 10(A)) [150]. In contrast, a group of PPTN neurons exhibited an increase in activity to reward cue presentation, and this activity was sustained throughout the task period. Some neurons showed stronger activity when the predicted reward was larger (Figure 10(B)), while others did not show any reward magnitude-dependent modulation (Figure 10(C)). Both types of neurons showed behavioral performance-related modulation, even before cue onset. Similar to the PPTN, a group of DRN neurons also showed stronger activity for larger-reward-predicting cue (Figure 10(E)). In addition, another group of DRN neurons exhibited the opposite firing pattern, that is, decreased activity for cue predicting a larger reward (Figure 10(G)). Unlike the PPTN, the DRN neurons with no significant reward modulation showed phasic responses to target presentation and slightly elevated activity during the fixation period (Figure 10(F)).

In the later phases of the trial, the monkeys received a juice reward. The dopaminergic neurons now exhibited a phasic burst or pause in activity immediately after cue-reward contingency reversal, in which the reward value was larger or smaller than expected, respectively, (Figure 10(A), dashed line). The PPTN neurons that showed tonic firing to the cue ceased firing around the time of reward delivery (Figures 10(B) and 10(C)) and were totally unresponsive to an unpredictably given reward. A different group of PPTN neurons, which did not modulate their activity in response to the cue, now exhibited a phasic burst to reward delivery (Figure 10(D)), and the response magnitude was correlated with the given reward value. Tonic-firing DRN neurons also showed a prolonged modulation of activity after reward delivery (Figures 10(E) and 10(G)). The reward-related modulation tended to be correlated with the modulation in activity during the fixation period. Notably, the changes in activity for large and small rewards tended to be in the opposite direction; for example, the postreward activity increased during large-reward trials, but it was inhibited during small-reward trials or vice versa. When there was a reward prediction error, just after cue-reward contingency reversal, the response of the reward delivery neurons of the PPTN (Figure 10(D)) and DRN (Figures 10(E) and 10(G)) faithfully followed the actual magnitude of the reward.

Some limitations of these extracellular recording studies in monkeys have to be considered. First, the PPTN and DRN are heterogeneous nuclei and contain various kinds of neurons. In our current experiments, however, the neurochemical identity of the recorded neurons was hard to determine. To date, we have not found a significant relationship between the firing pattern of the neurons and their neurophysiological characteristics, such as spike width, firing regularity, and recording site. Second, the PPTN/DRNs have massive reciprocal interconnections, not only with dopaminergic neurons but also with other brain areas; thus, the firing patterns of the neurons could be either input or output signals. While we found several types of representation, that is, tonic fixation and phasic reward modulation of PPTN neurons and positive and negative reward modulation of DRN neurons, the organization of these circuits and their interactions are hard to understand. With due consideration given to these methodological limitations, we believe that the present study contributes to our understanding of the role of neuromodulator systems in reinforcement learning and motivational behavioural control.

### 4.2. PPTN/DRN Neurons Relay the Tonic Reward Prediction Signal

A prominent feature of PPTN/DRN neuronal activity is its tonic modulation pattern, and these tonic firing patterns during the task period resemble the short-term memory of the reward prediction for the current trial. Computational models [151–155] of dopaminergic neuronal firing have noted similarities between the response patterns of dopaminergic neurons and the well-known learning algorithms, especially temporal difference reinforcement learning algorithms. However, there has been considerable debate regarding the circuit mechanisms underlying reward prediction error computation [154].

The temporal difference model uses fast-sustained excitatory reward prediction and delayed slow-sustained inhibitory signals in dopaminergic neurons to produce an onset burst to the cue followed by offset suppression to the reward. Previous studies have suggested that there are several structures that might send the tonic inhibitory reward prediction signals to dopaminergic neurons, such as the striosome [154, 155] and ventral pallidum [156]. However, the crucial missing link between the learning algorithm and the reported neuronal activity is the excitatory tonic input to dopaminergic neurons, which resembles the memory of the predicted reward value maintained until the actual reward delivery. The classical model supposed that the neurons in the striatum (the striosome) might provide both signals via direct and double-inhibition mechanisms to dopaminergic neurons. Our present findings suggest that a group of PPTN/DRN neurons could send a direct tonic excitatory component to dopaminergic neurons. How are these tonic signals from PPTN/DRN neurons converted to the phasic signals observed in the dopaminergic neurons? The simple and algorithm-matched model is the summation of the excitatory and inhibitory tonic signals, as follows. When the reward cue is presented, dopaminergic neurons receive a fast-sustained excitatory reward prediction signal, which we proposed, and a delayed slow-sustained inhibitory signal from the basal ganglia. DRN neurons can play either an excitatory or inhibitory role because the excitatory and inhibitory types of neurons are present, and serotonin exerts excitatory and inhibitory effects via several subtypes of serotonergic receptors [96]. As a result of summation, dopaminergic neurons exhibited transient excitatory and inhibitory signals timed at reward cue presentation and reward delivery, respectively. An alternative model for the computation suggests that the temporal differentiation of the tonic reward prediction signal, which increases at reward cue presentation and falls around the time of reward delivery, may produce the phasic signals of dopaminergic neurons. During the reward delivery phase, the inhibitory transients are summed with the excitatory actual reward signals by the other group of PPTN neurons, which we proposed, for the computation of the reward prediction error; thus, dopaminergic neurons produce no response when the reward prediction matches the actual one [14, 157].

Recent studies have emphasized the potential importance of the lateral habenula and rostromedial tegmental nucleus for the inhibition of dopaminergic neurons [158, 159]. Neurons in the lateral habenula are inhibited by a reward-predicting stimulus, but fire following a nonreward signal [160]. These structures are other possible candidates for the computation of the reward prediction error and are also interconnected with the PPTN and the DRN [65].

### 4.3. PPTN/DRN Neurons Relay the Task Motivation Signal

In addition to the reward prediction signal, an overlapping group of PPTN/DRN neurons showed task motivation-related activity modulation. The majority of PPTN neurons exhibited a tonic increase in activity regardless of its reward-related modulation. This tonic increase in activity occurred even before reward cue presentation, and part of these responses showed a significant dependency on the monkeys' performance of the task, such that stronger activity is observed during a good-performance epoch than during a poor-performance epoch. The recruitment of the PPTN in motivational control concurred with previous studies [30, 114, 161]. Conversely, task-related changes in DRN neurons included excitation and inhibition of activity. Furthermore, the reward-related modulation tended to be correlated with the initial task-related modulation, such that neurons with elevated activity exhibited stronger activity for a large reward than for a small reward. This observation suggests that DRN neurons encode correlated task and reward information, while PPTN neurons encode these signals independently.

### 4.4. PPTN/DRN Neurons Relay the Actual Reward Signal

In the reward delivery phase, PPTN and DRN neurons encode the “actual reward signal,” while dopaminergic neurons encode the “reward prediction error signal.” The actual reward signal is necessary information to compute the error between the predicted and actual reward; however, there are several differences between the actual reward signals of PPTN and DRN neurons. First, in the PPTN, two different groups of neurons encode the reward prediction and actual reward signals, while an overlapping group of DRN neurons encode both signals. Thus, PPTN neurons exhibited phasic burst firing only to reward delivery and were almost silent during the task period, while DRN neurons exhibited tonic firing both before and after reward delivery. Second, the actual reward responses of PPTN neurons were phasic, while DRN neurons exhibited a tonic modulation pattern that was sometimes sustained until just before the next trial. Third, PPTN neurons exhibited an increase in firing to large- and small-reward delivery, while DRN neurons exhibited an opposite response to these rewards.

These observations suggest that PPTN neurons encode a simple reward value, while DRN neurons encode rather more complex information. The correlated coding of task and reward signals by DRN neurons might be matched with the reported relationship of serotonin to impulsive behavior. One hypothesis is that DRN neurons integrate task-related reward prediction signals and actual received reward signals and have a role in time discounting for future rewards. A recent study in rats also reported that DRN neurons increased tonic firing while the rats waited for a reward, and this was related to the rats' waiting behavior [143]. Another hypothesis is that the actual reward signal of DRN neurons might be biased by the possible reward value for a rather long time scale (across blocks of trials). As shown above, even when the delivered magnitude of the reward was as predicted, some DRN neurons showed a decrease in firing to small-reward delivery; thus, DRN neurons might encode the error between the actual reward and the average of all possible options for rather a long time scale. Such patterns of relative reward value coding would be useful in comparing and selecting reward options, including reward value and time delay for receiving a reward.

Overall, the activity patterns of PPTN and DRN neurons were different from those of dopaminergic neurons, which are well known as the reward prediction error signal. Furthermore, the reward prediction and actual reward signals of PPTN/DRN neurons, which we proposed, are necessary signals for the computation of the reward prediction error and the appropriate action selection in a given situation. The different modulation patterns of the PPTN and DRN, together with the activity of dopaminergic neurons, reveal dynamic information processing between these different neuromodulator systems.
